# A Pilot Study on the Replacement of Fibrinogen with Fibrinogen Concentrates During Therapeutic Plasma Exchange with Mild to Moderate Bleeding Risk—A Comparison with Fresh Frozen Plasma and Albumin Replacement

**DOI:** 10.3390/jcm13247662

**Published:** 2024-12-16

**Authors:** Matej Zrimsek, Jakob Gubensek, Andreja Marn Pernat

**Affiliations:** 1 Department of Nephrology, University Medical Centre Ljubljana, Zaloska cesta 7, 1000 Ljubljana, Slovenia; 2 Faculty of Medicine, University of Ljubljana, Vrazov Trg 2, 1000 Ljubljana, Slovenia

**Keywords:** therapeutic plasma exchange, coagulation, fibrinogen concentrate, fresh frozen plasma, bleeding risk

## Abstract

**Background**: Therapeutic plasma exchange (TPE) removes coagulation factors and leads to depletion coagulopathy. The aim of the study was to compare hemostasis between TPE procedures without coagulation factor replacement (electrolyte group), the partial replacement of fibrinogen with fibrinogen concentrates (fibrinogen group) and partial coagulation factors replacement with fresh frozen plasma (partial FFP group). **Methods**: A total of 73 TPE procedures in patients with fibrinogen levels 1–2 g/L were divided into three study groups depending on clinically estimated bleeding risk. Standard coagulation and ROTEM^®^ tests were performed before and after TPE. **Results**: Fibrinogen levels before TPE (*p* = 0.88) and after TPE (*p* = 0.33) were comparable between the fibrinogen and partial FFP groups. INR and ROTEM^®^ parameters reflected moderately worse hemostasis after TPE with fibrinogen-only replacement compared to partial FFP replacement, which could result in increased bleeding risk. In the electrolyte group, most laboratory tests confirmed the most deranged hemostasis after TPE, as compared to fibrinogen or partial FFP replacement. A mild allergic reaction to FFP infusion was noted during one TPE. No clinically significant bleeding occurred in any of the study groups. **Conclusions**: Fibrinogen concentrate supplementation and partial FFP replacement can both maintain fibrinogen levels > 1 g/L after TPE, but modest differences in classical coagulation tests and bedside ROTEM^®^ tests favor FFP replacement (NCT03801135).

## 1. Introduction

Therapeutic plasma exchange (TPE) is an extracorporeal procedure in which the patient’s plasma is removed and replaced with a substitute fluid [1]. As it eliminates the patient’s antibodies and immune complexes, it is used to treat various autoimmune diseases, the humoral rejection of solid organ transplants and to desensitize highly sensitized patients prior to transplantation [2]. In addition to antibodies, coagulation factors, albumin and other plasma components are also removed. The replacement fluid during TPE consists of 3% albumin in an electrolyte solution, fresh frozen plasma (FFP) or a mixture of both [3], depending on the pathophysiology of the disease being treated and the patient’s bleeding risk [4]. FFP should be used as the sole or predominant replacement solution in thrombotic thrombocytopenic purpura, in patients with advanced liver failure and when TPE is performed before major surgery [3,4]. The use of FFP is associated with an increased risk of adverse reactions [5,6,7]; also, the additional citrate contained in FFP increases the likelihood of significant metabolic alkalosis after TPE [8,9]. Therefore, whenever possible, an electrolyte solution with albumin as a replacement fluid is used and FFP is avoided.

As TPE treatment usually involves repeated procedures with a relatively short interval between procedures (especially in more severe cases of disease) [3], the use of electrolyte replacement fluid leads to a reduction in the concentration of clotting factors which increases the risk of bleeding (i.e., depletion coagulopathy) [10,11,12,13,14]. Studies have shown that the concentration of fibrinogen and other clotting factors decreases significantly during TPE without FFP replacement. It is unclear how to best assess apheresis-related bleeding risk due to depletion coagulopathy and how to decide on the need for clotting factor replacement, as both pro- and anticoagulant factors are removed. Nevertheless, the fibrinogen level is traditionally used as a marker. It is probably more clinically relevant than standard coagulation tests, which are usually normal before repeated TPEs, while fibrinogen has one of the longest recovery times among coagulation factors and does not fully recover to baseline even after 48–96 h after the last TPE [3,4,14,15,16,17]. Traditionally, FFP is used as part of the replacement fluid (at a dose of 10–20 mL/kg body weight) when fibrinogen levels are borderline and the patient has increased bleeding risk. In this study, we postulate that fibrinogen concentrate replacement instead of partial FFP replacement may be sufficient in this situation to reduce bleeding risk, since the concentrations of most other clotting factors with shorter plasma half-lives recover faster than fibrinogen after a TPE procedure by synthesis from the liver [6,15,18].

We conducted a pilot study on the replacement with fibrinogen concentrate instead of partial FFP replacement in patients with borderline fibrinogen levels before TPE and mildly increased bleeding risk. We compared hemostatic tests during TPE procedures with fibrinogen concentrate replacement and compared them with partial FFP replacement and standard replacement solutions (electrolytes and albumin).

## 2. Materials and Methods

### 2.1. Study Design

**Figure 1:**
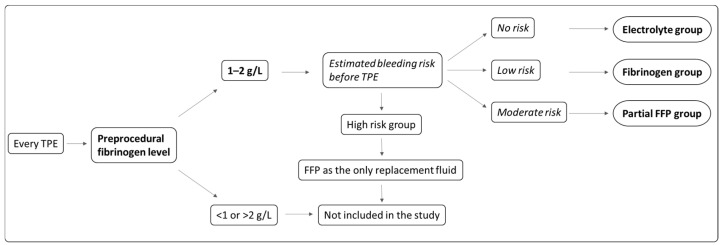
Treatment algorithm and choice of replacement solution during TPE based on fibrinogen level and estimated bleeding risk (see also Table 1).

This was a non-randomized, controlled interventional study, performed between October 2018 and March 2023 at the University Medical Centre Ljubljana. Individual TPE procedures, performed in adult patients with an indication for intensive TPE treatment, were included in one of the study groups based on a protocol described below. TPE procedures performed in each individual patient could be enrolled in different groups. The exclusion criteria were as follows: known coagulation abnormalities including significant liver disease, anticoagulant therapy, an indication for FFP as the only replacement solution, pregnancy and acute hypertriglyceridemic pancreatitis. Each TPE procedure was assigned to one of three study groups based on the replacement solution prescribed during the TPE: electrolyte solution (electrolyte group), electrolyte solution with fibrinogen concentrate infused after TPE (fibrinogen group) or electrolyte solution with partial FFP replacement (partial FFP group). The replacement solution was prescribed based on the pre-TPE fibrinogen level and clinically estimated bleeding risk (see Figure 1 and Table 1). A wide range of coagulation tests were performed before and after TPE and compared between the three groups. The study was conducted in accordance with the Declaration of Helsinki, approved by the National Medical Ethics Committee (No. 0120-214/2018/4) and registered at ClinicalTrials.gov (No. NCT03801135). Written informed consent was obtained from all participants prior to enrollment in the study.

### 2.2. Therapeutic Plasma Exchange (TPE) Procedure

Peripheral veins, jugular or femoral central venous catheters or arteriovenous fistula were used for vascular access. Membrane TPEs were performed using a Multifiltrate system device with a P2 Dry plasma filter (both Fresenius SE & Co. KGaA, Bad Homburg, Germany). One estimated plasma volume was exchanged per procedure, which was calculated based on of the patient’s weight and hematocrit value. Regional anticoagulation was performed with 8% sodium citrate (prepared by the hospital pharmacy), followed by intravenous calcium replacement via the venous line with 1 M CaCl_2_ at a dose adjusted to the patient’s ionized calcium level.

### 2.3. Study Groups and Replacement Solution

If the fibrinogen level before TPE was between 1 and 2 g/L and the patient was not at high risk of bleeding, the TPE procedure was included in the study. The study group and the replacement solution were determined by the attending physician and roughly followed the scheme shown in Figure 1. The criteria for the assessment of bleeding risk are listed in Table 1 and the exact value of pre-TPE fibrinogen itself (in the range of 1–2 g/L range) was also taken into account. In summary, the risk of bleeding was assessed by the attending physician, who analyzed all relevant clinical data, including time after invasive procedures, patients’ antithrombotic therapy and laboratory results.

In case that the patient did not have increased bleeding risk, TPE procedure was performed with a mixture of an electrolyte solution (Duosol, B. Braun, Melsungen, Germany) and 20% albumin (Octapharma, Lachen, Switzerland) added to a calculated albumin concentration of about 30 g/L as a replacement solution (electrolyte group). In case that the patient had a low bleeding risk, the TPE procedure was performed with the same replacement solution, with an additional infusion of a fibrinogen concentrate (Haemocomplettan, CSL Behring, Marburg, Germany or Fibrema, Octapharma, Lachen, Switzerland) administered intravenously after the procedure (fibrinogen group). The dose of fibrinogen concentrate was calculated with the aim of achieving a fibrinogen value of >1 g/L after TPE (considering fibrinogen values before TPE, the expected fibrinogen drop for about 60% during TPE and the patient’s weight) using the following formula $\frac{\left(desired\;rise\;of\;fibrinogen\;in\;\frac{g}{L}\;\right)\times (patients\;weight\;in\;kg)}{17\;\frac{L}{kg}\;}$. In the case that the patient had a moderate bleeding risk, the TPE procedure was performed with a mixture of albumin solution and FFP (approximately 10–15 mL/kg body weight, partial FFP group). If the patient had a high risk of bleeding, only FFP was used as a replacement solution and the TPE procedure was not included in the study.

### 2.4. Laboratory Tests

The blood samples for the laboratory tests were taken immediately before and after each procedure (and after the fibrinogen infusion in the fibrinogen group) via the vascular access. Blood counts were obtained from EDTA blood samples, and the citrated blood samples were used for the analysis of standard coagulation tests (prothrombin time with international normalized ratio (INR), activated partial thromboplastin time (aPTT), fibrinogen level) and rotational thromboelastometry (ROTEM^®^). Standard coagulation tests were determined with ACL TOP 700 device (Instrumentation Laboratory, Bedford, MA, USA). For prothrombin time, a Recombiplastin Hemosil was used, for aPTT and aPTT-SP, Hemosil, and for fibrinogen concentration, Hemosil Q.F.A. thrombin bovine kit (all Werfen, Barcelona, Spain). For determining the fibrinogen level, the Clauss method was used. Additionally, we also measured factor XIII level (antigen measurement, also a Hemosil reagent). The ROTEM^®^ test was performed according to the manufacturer’s protocol (Pentapharm, Munich, Germany). The EXTEM test with clotting time (CT), clot formation time (CFT) and maximum clot firmness (MCF), the INTEM test with CT, CFT and MCF and the FIBTEM test with CT and MCF were performed.

### 2.5. Statistical Analysis

The statistical analyses were performed with SPSS Statistics 27 (IBM, Armonk, NY, USA) and some of the graphs were produced by Jamovi ver. 2.5.2.0 (retrieved from https://www.jamovi.org, accessed on 1 September 2024). Means and standard deviations are given for normally distributed parameters; medians and interquartile ranges are given for non-normally distributed parameters. To compare the values before and after the TPE within the groups, a paired Student’s T-test or a Wilcoxon signed rank test was used, depending on the distribution. ANOVA with Gabriel or Games–Howell post hoc test was used to compare pre- and post-TPE values, as well as the absolute and relative differences between pre- and post-TPE values between the groups. Linear regression was used to predict post-TPE values from pre-TPE values. *p*-values were considered significant at <0.05.

## 3. Results

The study analyzed results from 73 TPE procedures performed on 40 patients with an average age of 58 ± 15 years and an average weight of 76.4 ± 14.9 kg. Twenty-six patients were male (65%) and fourteen (35%) were female. The indications for TPE are listed in Table 2.

In 42 (58%) TPE procedures, the patients did not receive any coagulation factors supplementation during or after TPE (electrolyte group). After 12 (16%) TPE procedures, fibrinogen concentrate was administered after TPE (fibrinogen group), most often with 2 g of fibrinogen concentrate (on average 1.83 g). In 19 (26%) TPE procedures included in the study, FFP was a part of the replacement fluid (partial TPE group); the volume of FFP used was between 500 and 1500 mL.

The number of TPEs per patient varied between 5 and 15, but most often 5–7 repetitive TPE procedures were performed. Different TPE procedures from the same patient were included in different study groups as per protocol regarding the assessed bleeding risk. Supplementary Table S1 represents additional data regarding the frequencies of the sequential number of included TPEs and the baseline values of standard coagulation tests. Many TPEs were not included in the analysis (this was mostly because of their preprocedural fibrinogen value, but also because of the high bleeding risk and the inability of the laboratory to complete all of the planned tests at the time of TPE).

The mean hemoglobin concentrations before TPE were the highest in the partial FFP group: 110 ± 17 g/L in the partial FFP group, 102 ± 13 g/L in the electrolyte group and 99 ± 13 g/L in the fibrinogen group—the difference was borderline statistically significant (*p* = 0.050). The values after TPE did not differ significantly between the groups (*p* = 0.083) and increased on average for 1 ± 7 g/L, which was not significant (*p* = 0.21).

The mean platelet count before TPE was significantly lower in the partial FFP group (163 ± 38 × 10^9^/L) vs. the electrolyte group (199 ± 48 × 10^9^/L, *p* < 0.023) and it was also lower after TPE (156 ± 35 × 10^9^/L vs. 187 ± 49 × 10^9^/L, *p* = 0.051). The decrease in platelets during TPE was significant (*p* < 0.001), with an average decrease of 11 ± 17 × 10^9^/L.

**Figure 2:**
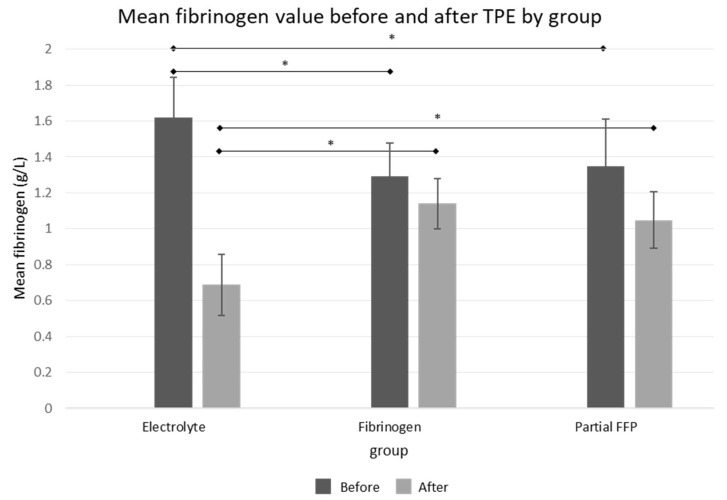
Mean fibrinogen values before and after the TPE procedure in all three groups. See Supplementary Table S2 for exact values and comparisons. *—significant differences (*p* < 0.05 by ANOVA post hoc test) between groups.

The comparison of baseline fibrinogen levels between groups showed that pre-TPE levels were significantly lower in the fibrinogen and partial FFP groups (1.29 ± 0.18 g/L in fibrinogen group and 1.34 ± 0.02 in partial FFP group vs. 1.62 ± 0.22 g/L in the electrolyte group, *p* < 0.001; see Figure 2 and Supplementary Table S2), probably reflecting the attending physician’s tendency to administer fibrinogen/FFP at lower fibrinogen levels. A comparison between the fibrinogen and partial FFP groups showed that fibrinogen levels were comparable not only before TPE (*p* = 0.875) but also after TPE (*p* = 0.334), suggesting that the substitution of fibrinogen with fibrinogen concentrate was comparable to partial FFP replacement and both were able to maintain fibrinogen levels above 1 g/L after TPE (see Supplementary Figure S1).

**Figure 3:**
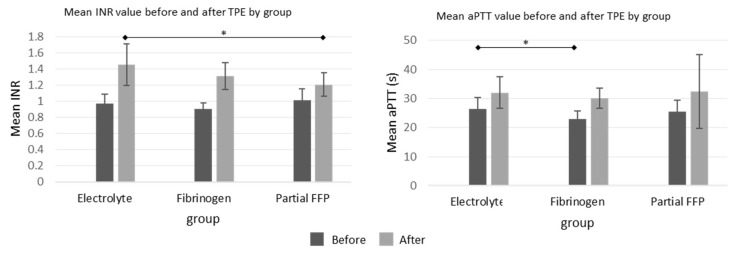
Mean INR and aPTT values before and after the TPE procedure in all three groups. See Supplementary Table S2 for exact values and comparisons. *—significant differences (*p* < 0.05 by ANOVA post hoc test) between the groups.

The differences before and after TPE procedures in APTT values were comparable between the groups, but the increase in INR values was more pronounced in the electrolyte and fibrinogen groups than in the partial FFP group, which is shown graphically in Figure 3, while all values with detailed analysis are listed in Supplementary Table S2.

**Figure 4:**
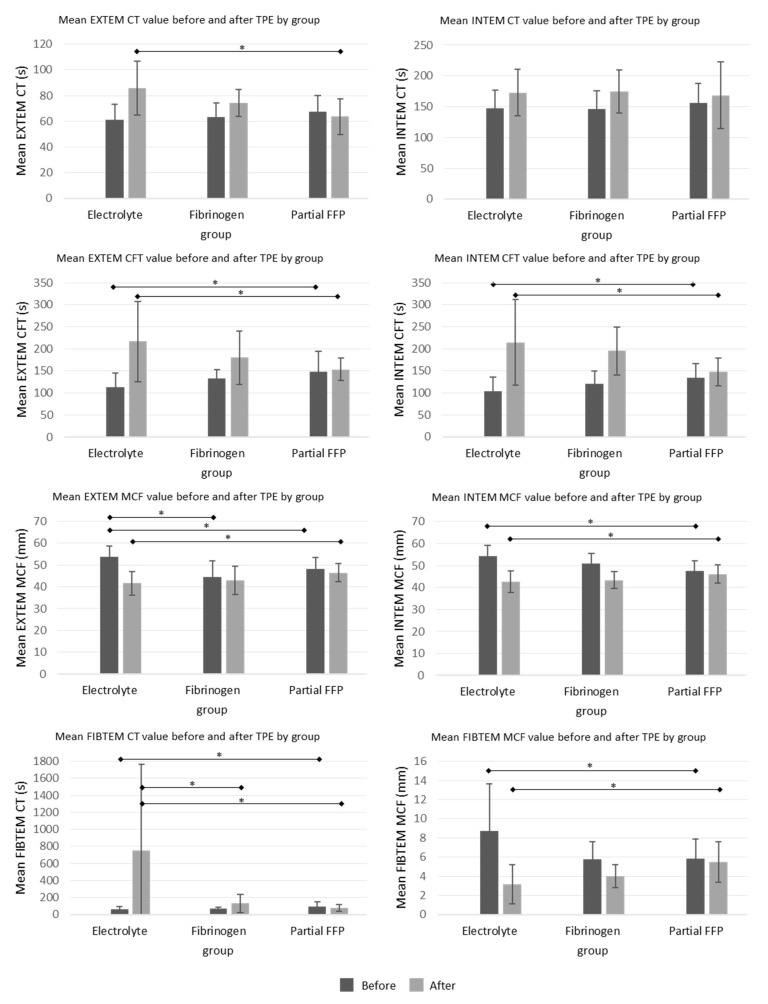
Mean ROTEM values before and after the TPE procedure in all three groups. See Supplementary Table S2 for exact values and comparisons. *—significant differences (*p* < 0.05 by ANOVA post hoc test) between the groups.

**Figure 5:**
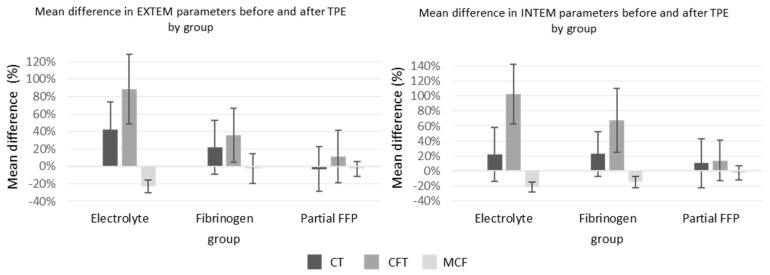
Mean percent change between before and after TPE values of EXTEM and INTEM in all three groups. See Supplementary Table S2 for exact values and comparisons.

The ROTEM parameters are shown graphically in Figure 4. Among them, INTEM-CT (similar to aPTT) was comparable between groups, while EXTEM-CT after TPE (similar to INR) was the most prolonged in the electrolyte group, especially compared to the partial FFP group. The mean values of INTEM and EXTEM-CFT in the partial FFP group were the longest before and shortest after TPE in comparison to other groups. Therefore, the partial FFP group showed the smallest deterioration in hemostasis during TPE (see Figure 4 and Figure 5). The values after TPE in the fibrinogen group were also better than those in the electrolyte group, proving the benefit of fibrinogen supplementation. Mean percentage differences between before and after values in EXTEM and INTEM compared in all the groups are represented in Figure 5. As expected, the INTEM and EXTEM-MCF decrease was the highest in the electrolyte group where the FIBTEM-CT times were also extremely prolonged due to the extremely low fibrinogen concentrations after TPE, whereas the FIBTEM-CT times in the partial FFP and fibrinogen groups did not differ significantly after TPE and their prolongation during TPE was also comparable. FIBTEM-MCF decreased significantly by about 60% in the electrolyte group and by 28% in the fibrinogen group, while there was almost no change in the partial FFP group. The difference between the partial FFP and fibrinogen groups is probably due to the partial replacement of factor XIII by FFP (see Supplementary Table S2).

Regarding complications, one immobile patient with Guillain–Barre syndrome developed femoral vein thrombosis at the site where the catheter was inserted. This patient did not receive fibrinogen concentrate or fresh frozen plasma during his TPE treatments. A mild allergic reaction to FFP infusions was noted during one TPE. No clinically significant bleeding occurred.

## 4. Discussion

Our pilot study on fibrinogen replacement in TPE treatment showed comparable fibrinogen levels after TPE in the fibrinogen concentrate and partial FFP replacement groups, confirming that both are able to maintain fibrinogen levels above 1 g/L after TPE, which should prevent excessive bleeding risk. As expected, fibrinogen concentrate does not replace other coagulation factors as FFP does, resulting in some modest differences in classical hemostatic tests (greater increase in INR) as well as in some components of the ROTEM^®^ bedside test (mainly EXTEM and INTEM-CFT). These results reflect moderately worse hemostasis immediately after TPE with pure fibrinogen replacement and could lead to an increased risk of bleeding. It is likely that these differences slowly disappear hours after TPE, as new coagulation factors are synthesized. On the other hand, as expected, patients who did not receive coagulation factor replacement during TPE (the electrolyte group) had worse hemostatic test results than patients with fibrinogen or partial FFP replacement.

It is known that TPE affects hemostasis on several levels [14]. In general, there is a decrease in platelet count during TPE [10,12,13,19], which we also observed in all three groups. In addition, Feuring et al. also published an article describing how plasma donation impairs the hemostatic capacity of platelets [20]. It has been reported that even isovolumic TPE with FFP alters ROTEM^®^ parameters, especially clotting time in the intrinsic and extrinsic pathways [21]. The effect of TPE on the coagulation cascade is complex, as both procoagulant and anticoagulant factors are removed and different factors have different recovery times [6]. Due to the different recovery times, the ratio of coagulation factors changes with repeated TPE procedures. This makes comparative studies analyzing the effect of different replacement solutions on hemostatic parameters more difficult. Fibrinogen and factor XIII have the longest recovery time (96–120 h), and their baseline values vary from patient to patient [6,15]. Therefore, maintaining adequate fibrinogen levels during TPE treatment is considered essential. Fibrinogen concentrate is commonly used in clinical practice for surgical bleeding and has a better safety profile than fresh frozen plasma [18]. Therefore, we hypothesized that it might also be useful in depletion coagulopathy induced by repeated TPE treatments.

Our comprehensive hemostatic evaluation of the three replacement solutions also included a ROTEM^®^ analysis. EXTEM and INTEM MCF values before TPE were significantly higher in the electrolyte group, probably due to higher fibrinogen levels, as MCF is mainly influenced by fibrinogen concentration, factor XIII level and platelet count [22]. Although fibrinogen levels were significantly lower in the electrolyte group after TPE, EXTEM and INTEM MCF levels were not as low as would be expected compared to the fibrinogen or partial FFP group. This is probably due to the significant involvement of thrombocytes in clot formation and the low factor XIII levels in all groups. If the effect of platelets on clot formation is excluded, which is the case with the FIBTEM test, then the changes observed with FIBTEM CT and MCF between the groups become much more pronounced. On the other hand, there was no statistical difference in fibrinogen levels before and after TPE between the fibrinogen and partial FFP groups, while factor XIII was significantly higher in the partial FFP group after TPE. Higher factor XIII levels, which are important for better clot polymerization, and higher levels of other coagulation factors due to their partial replacement probably explain the best CFT results in both EXTEM and INTEM tests in the partial FFP group compared to the other two groups. Chuliber et al. postulated that acquired factor XIII deficiency after TPE without coagulation factor replacement could be a cause of postoperative bleeding [23]. Their patients also had decreased fibrinogen levels after TPE, and none of them received fibrinogen concentrate. Some received factor XIII concentrate, after which the bleeding stopped, but in some patients, the bleeding stopped even without clotting factor replacement. Our patients had low factor XIII levels which were still mostly above 15%, which should be sufficient to prevent spontaneous bleeding, but could be a contributing factor to possible postoperative bleeding. Further studies with factor XIII replacement in combination with fibrinogen replacement in TPE are needed.

Over the past decade, the use of the ROTEM^®^ test at the bedside in patients with perioperative or trauma-related bleeding and the application of clinical algorithms based on the results has increased significantly [22,24,25]. The algorithms were developed for the case of active bleeding, which is not the case for most patients referred to TPE. Nevertheless, many patients requiring TPE may be at moderate risk of bleeding. According to the ROTEM^®^ test manufacturer’s recommendations, patients with an INTEM or EXTEM-MCF between 40 and 45 mm are at risk of bleeding, while an MFC between 30 and 39 mm represents a high risk of bleeding. A CFT between 160 and 220 s indicates a reduced hemostatic reserve and a CFT between 221 and 300 s indicates a bleeding risk. For surgical procedures, clinicians usually aim for an MCF of at least 40 mm and a CFT below 300 s [26]. According to this interpretation, most patients in our study had, on average, adequate ROTEM^®^ values after TPE, regardless of the type of replacement fluid. However, many of our patients already had a low hemostatic reserve before TPE. During surgical procedures, coagulation factors can be additionally depleted and therefore the ROTEM^®^ tests should be repeated. In the case of bleeding, clinicians aim for a complete normalization of ROTEM^®^ results. Therefore, in the case of bleeding, our patients in all three groups should have received an additional fibrinogen concentrate after TPE according to the algorithms, as all had suboptimal FIBTEM results.

In summary, although the results of the fibrinogen group were better than those of the electrolyte group, indicating increased safety for patients receiving fibrinogen concentrate, they were not fully comparable to the partial FFP group, at least not when taken immediately after TPE as in our study. Further studies looking at coagulation tests performed a few hours after TPE to compare fibrinogen and partial FFP replacement could show comparable results between the two groups, as many coagulation factors have shorter half-life than fibrinogen. Such a study should be conducted in the future and would further confirm the safety of fibrinogen-only replacement in patients at a moderate risk of bleeding.

Our study has some limitations. It was a controlled, but not randomized study, which limits the firmness of its conclusions. The selection of replacement fluid was based on clinically assessed preprocedural bleeding risk. Further variability between the groups was introduced by the discretion of the physician prescribing the type of replacement fluid to consider the exact preprocedural fibrinogen level within the 1–2 g/L interval. Although this probably increased patient safety, it decreased the quality of the study, because significantly higher fibrinogen levels were present in the electrolyte group before TPE. Furthermore, we were unable to assess the clinical significance (bleeding events) of the measured hemostatic parameters due to the small number of patients included. As the rate of bleeding complications reported in the literature for TPE treatments is very low, such an analysis would require a very large number of patients.

## 5. Conclusions

In conclusion, our results show that pure fibrinogen replacement during intensive TPE treatment is not fully equivalent to clotting factor replacement by FFP, but still significantly improves hemostasis after TPE. The use of FFP is probably indicated as partial or sole fluid replacement in TPE when TPE is performed immediately prior to major surgery, while fibrinogen replacement is probably sufficient in patients with mild to moderate clinical bleeding risk. Further studies with coagulation tests performed a few hours after TPE would be informative in this regard and studies with factor XIII replacement, which might further improve ROTEM-CFT and MCF values, are also needed.

## Figures and Tables

**Table 1:** Criteria for the assessment of clinical bleeding risk in study patients.

High bleeding risk—not included in the study:
≤14 days after major intracranial or neuraxial procedure ≤7 days after other major surgical procedure (thoracic, cardiac, abdominopelvic orthopedic)
≤3 days after colonoscopy with polypectomy, kidney or liver biopsy; minor abdominal, thoracic or orthopedic procedures
Acute diffuse alveolar bleeding
**Moderate bleeding risk:**
15–21 days after major intracranial or neuraxial procedure 8–14 days after other major surgery (thoracic, cardiac, abdominopelvic, orthopedic)
4–7 days after kidney or liver biopsy, minor abdominal, thoracic or orthopedic procedures
≤3 days after lumbar puncture or removal of a large arterial catheter/sheath
Suspected alveolar hemorrhage
**Low bleeding risk:**
22–28 days after major intracranial or neuraxial procedure 15–21 days after other major surgery (thoracic, cardiac, abdominopelvic, orthopedic)
8–10 days after a kidney or liver biopsy; minor abdominal, thoracic or orthopedic procedures
4–7 days after a lumbar puncture
**No bleeding risk:**
Not fulfilling any of the above criteria

**Table 2:** Indications for therapeutic plasma exchange treatment in study patients.

Indications	Number of Patients
Humoral rejection of transplanted kidney	11
Guillain–Barre syndrome	6
Myasthenia gravis	5
Antineutrophil cytoplasmic antibody (ANCA) vasculitis	4
Neuromyelitis optica	3
IgA vasculitis with rapidly progressive glomerulonephritis	2
Cryoglobulinemic vasculitis	2
Monoclonal gammopathy with renal significance	1
Focal segmental glomerulosclerosis	1
Chronic inflammatory demyelinating polyneuropathy	1
Anti-glomerular basement membrane disease	1
Anti-glomerular basement membrane disease with ANCA vasculitis	1
Transverse myelitis	1
Systemic sclerosis with symptomatic hypergammaglobulinemia	1

## Data Availability

All relevant data are incorporated into the article.

## Supplementary Materials

The following supporting information can be downloaded at https://www.mdpi.com/article/10.3390/jcm13247662/s1, Supplementary Table S1: The frequencies of TPEs by their sequence number within a course of TPE treatments; Supplementary Table S2: Mean fibrinogen, factor XIII, INR, aPTT and ROTEM values before and after TPE procedure in all three groups; Supplementary Figure S1: Linear regression analysis of fibrinogen levels before and after TPE by groups.
