# Supplementary figures and images for: A Pilot Study on the Replacement of Fibrinogen with Fibrinogen Concentrates During Therapeutic Plasma Exchange with Mild to Moderate Bleeding Risk—A Comparison with Fresh Frozen Plasma and Albumin Replacement
Source: J Clin Med. 2024 Dec 16;13(24):7662. doi: 10.3390/jcm13247662 (PMC11676064; doi:10.3390/jcm13247662)

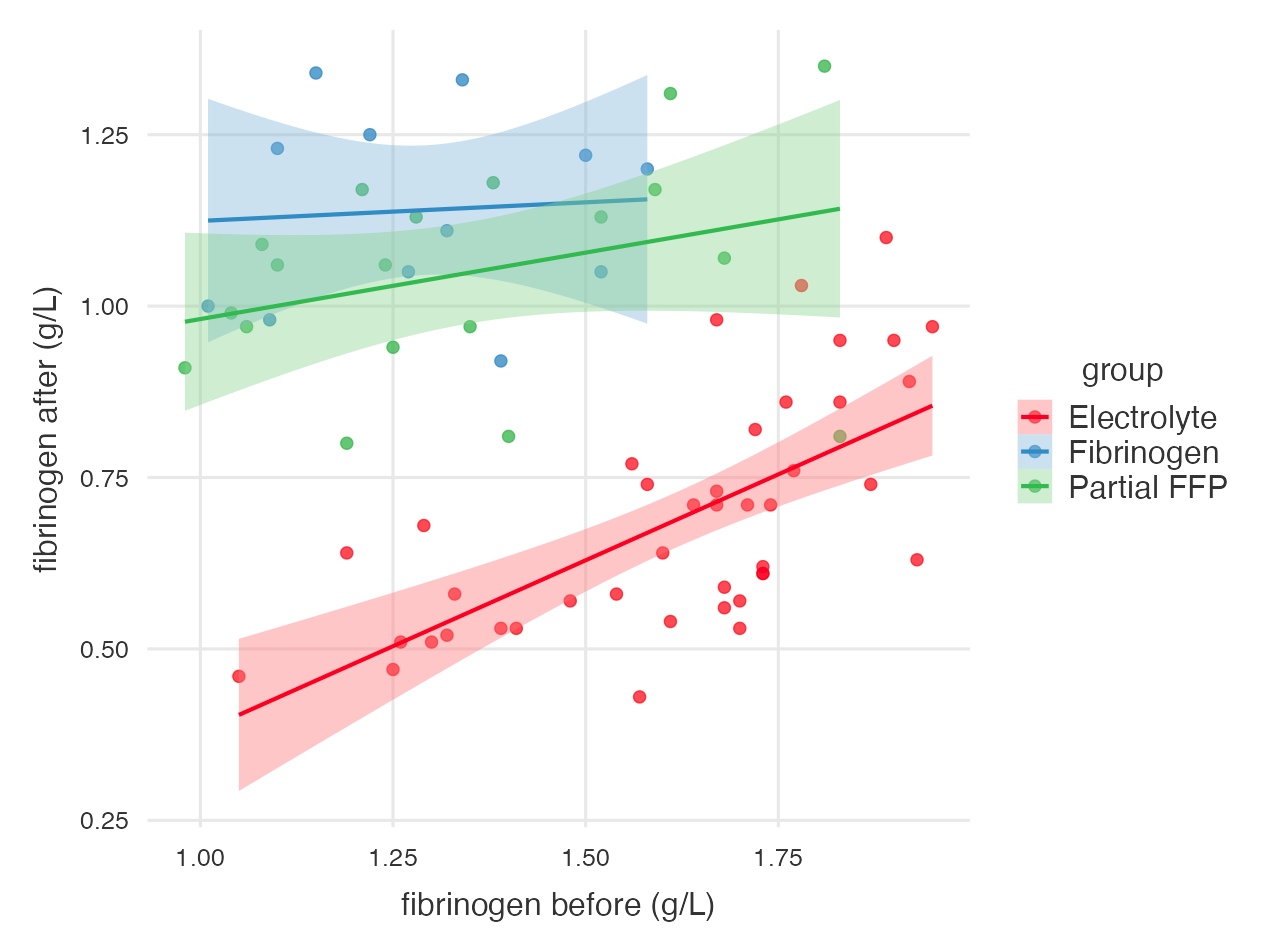

Supplement: Supplementary file 1 [file jcm-13-07662-s001.zip › Fib-by-group.tiff]
